# SOCIAL DETERMINANTS OF HEALTH AND AGING: DISPARITIES AND BARRIERS TO ORAL HEALTH CARE AMONG OLDER ADULTS IN ALABAMA

**DOI:** 10.1093/geroni/igad104.2474

**Published:** 2023-12-21

**Authors:** Nathan Smith, Stacey Cofield, Raquel Mazer

**Affiliations:** University of Alabama School of Dentistry, Birmingham, Alabama, United States; University of Alabama at Birmingham, School of Public Health, Birmingham, Alabama, United States; University of Alabama at Birmingham, School of Dentistry, Birmingham, Alabama, United States

## Abstract

Limited data exists on the oral health status of the older adult population of Alabama; improved surveillance programs are needed for estimations and planning. Aim: This study compares gender, racial, and economic differences in barriers to oral healthcare among older adults in Alabama. Methods: Self-reported, voluntary surveys including sociodemographics, oral care behaviors, and care utilization were completed along with intraoral screenings at senior centers and senior living communities. Univariate analyses were conducted using Chi-Square or Fisher’s Exact test, p< 0.05 considered meaningful. Results: 357 respondents were assessed: mean age 73.4 (SD 8.4), 70.3% female, 57.1% Black/African American (AA), 42.9% Caucasian (Ca), 88.8% sole source of income Social Security/Disability. Ca were more likely than AA to have dental insurance (65.0 vs 51.5%, p=0.013), and more likely to have had a cleaning/check-up (20.9 vs 12.3%, p=0.029). Those with income <$1500/month who received dental care were more likely to receive care for dentures compared to those with higher income (17.1 vs 8.0%, p=0.030). Those still driving who received dental care were more likely to have had a cleaning/check-up compared to those not still driving (20.9 vs 9.2%, p=0.025). Untreated tooth decay was detected in 25% of those with remaining dentition and was more common among AA than Ca (57.4 vs 44.4%, p=0.035). Conclusions: The preliminary analysis points to the need to further explore access to oral health services for older Alabamians living independently. Further data is needed for planning and policy making. Barriers to care were not explored for those who are homebound.

